# Rapeseed and milk protein exhibit a similar overall nutritional value but marked difference in postprandial regional nitrogen utilization in rats

**DOI:** 10.1186/1743-7075-8-52

**Published:** 2011-07-25

**Authors:** Claire Boutry, Hélène Fouillet, François Mariotti, François Blachier, Daniel Tomé, Cécile Bos

**Affiliations:** 1 INRA, CNRH-IdF, UMR914 Nutrition Physiology and Ingestive Behavior, F-75005 Paris, France; 2 AgroParisTech, CNRH-IdF, UMR914 Nutrition Physiology and Ingestive Behavior, F-75005 Paris, France

**Keywords:** dietary protein, postprandial metabolism, nutritional quality, tissue protein synthesis

## Abstract

**Background:**

Rapeseed is an emerging and promising source of dietary protein for human nutrition and health. We previously found that rapeseed protein displayed atypical nutritional properties in humans, characterized by low bioavailability and a high postprandial biological value. The objective of the present study was to investigate the metabolic fate of rapeseed protein isolate (RPI) and its effect on protein fractional synthesis rates (FSR) in various tissues when compared to a milk protein isolate (MPI).

**Methods:**

Rats (n = 48) were given a RPI or MPI meal, either for the first time or after 2-week adaptation to a MPI or RPI-based diet. They were divided in two groups for measuring the fed-state tissue FSR 2 h after the meal (using a flooding dose of ^13^C-valine) and the dietary N postprandial distribution at 5 h (using ^15^N-labeled meals).

**Results:**

RPI and MPI led to similar FSR and dietary nitrogen (N) losses (ileal and deamination losses of 4% and 12% of the meal, respectively). By contrast, the dietary N incorporation was significantly higher in the intestinal mucosa and liver (+36% and +16%, respectively) and lower in skin (-24%) after RPI than MPI.

**Conclusions:**

Although RPI and MPI led to the same overall level of postprandial dietary N retention in rats (in line with our findings in humans), this global response conceals marked qualitative differences at the tissue level regarding dietary N accretion. The fact that FSR did not however differed between groups suggest a differential modulation of proteolysis after RPI or MPI ingestion, or other mechanisms that warrant further study.

## Background

It's renewed interest to investigating plant protein sources for human nutrition because of the growing demand for high-quality healthy food coupled with in the context of reasoning policy changes for sustainable and healthy dietary choices [1-3]. We recently explored the potential of rapeseed protein (also referred to as canola protein), an emerging protein source for human nutrition [4-6]. Rapeseed protein displays an interesting amino acid (AA) profile because none of the indispensable AA is limiting with respect to human requirements. This is fairly unique for a vegetable protein, as are the high levels of sulfur AA determined [7]. Of note, rapeseed protein also has a potential to limit the onset of the metabolic syndrome [5,6].

The global nutritional value of rapeseed protein *in vivo *is equivalent to that of other legume sources of good nutritional quality, such as soy, pea or lupin [4,8]. However, rapeseed protein presents an atypical digestive and metabolic fate when compared to these legume sources or to other dietary proteins of animal origin. The digestibility of rapeseed protein is relatively poor in humans or pigs because of the presence of hydrolysis-resistant fractions [4,8-10]. However, this relatively poor digestibility is largely compensated for, by the very high biological value of rapeseed protein, as demonstrated in humans [4]. Lastly, rapeseed protein has been shown to display an atypical pattern of postprandial tissue N retention when compared to other protein sources in humans characterized by a predominantly splanchnic retention at the expense of the peripheral area (Fouillet *et al*. unpublished results), as estimated using a previously developed 13-compartment model [11,12]. These findings suggest that rapeseed protein might exert tissue-specific effects on postprandial protein kinetics. To test this hypothesis, we studied the differential influence of rapeseed protein and milk protein on both tissue protein synthesis rates and postprandial dietary N accretion in rats. The rats under study either received the protein sources for the first time in the test meal, or after the protein source had been supplied in their background diet for 2 weeks, the aim being to elucidate whether a potential adaptation to rapeseed protein could change their postprandial responses.

## Methods

### Animals

All experiments were carried out in accordance with the guidelines of the French Committee for Animal Care and the European Convention on Vertebrate Animals Used for Experimentation. Forty-eight male Wistar rats (Harlan, Horst, The Netherlands) weighing 175-200 g at the beginning of the experiment were housed in individual stainless steel cages in a ventilated room under a controlled temperature (22 ± 1°C) and a 12:12-h light-dark cycle (lights on: 20:00-08:00). They were acclimatized to the conditions prevailing in the animal facility and fed a chow diet (Scientific Animal Food and Engineering, Villemoisson sur Orge, France) for 6 days. The animals were allowed free access to water throughout the experimental period.

### Dietary adaptations

**Figure 1:**
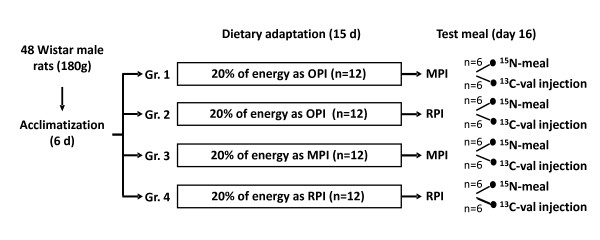
**Experimental protocol**.

On day 7, the rats were assigned randomly to one of four groups (n = 12 per group, Figure 1). Initial body weights did not differ between the groups. For 15 days, each group received a diet supplying 20% of energy as protein, 30% as fat and 50% as carbohydrate. The diets only differed in terms of their protein source: milk protein isolate (MPI), rapeseed protein isolate (RPI) or a combination of other protein isolates consisting of 33% wheat protein, 33% soy protein and 33% fish protein (OPI). The latter diet was only given to two of the four groups (Figure 1, Groups 1 and 2) that received only MPI or RPI as a meal on the last day (to study the acute effect of these protein sources), whereas the other two groups (Figure 1, Groups 3 and 4) received MPI or RPI all along the experimental period.

The experimental diets were prepared by the Atelier de Production des Aliments Expérimentaux (INRA, Jouy-en-Josas, France) (Table 1). The three diets were rendered isonitrogenous by adjusting for the variations in crude protein contents of the different isolates (MPI: 80%; RPI: 93%; Fish protein isolate: 89%; soy protein isolate: 89% and wheat protein isolate: 77%). Account was also taken of the small amounts of fats and/or carbohydrate present in protein isolate powders. The indispensable AA composition of the three diets also differed: the MPI diet was richer in indispensable AA (470 mg.g^-1 ^of protein), and in particular in branched-chain AA and tyrosine. RPI contained higher levels of sulfur AA and threonine.

**Table 1:** Compositions of the experimental diets based on MPI, RPI or a mix of OPI

	OPI	MPI	RPI
Milk protein isolate (g.kg^-1^)	-	249.1	-
Rapeseed protein isolate (g.kg^-1^)	-	-	216.9
Fish protein isolate (g.kg^-1^)	74.0	-	-
Soy protein isolate (g.kg^-1^)	74.0	-	-
Wheat protein isolate (Gluten) (g.kg^-1^)	85.5	-	-
Starch (g.kg^-1^)	483.5	486.6	493.9
Sucrose (g.kg^-1^)	49.4	33.8	50.5
Soy oil (g.kg^-1^)	131.4	127.7	134.2
Minerals (AIN-93M) (g.kg^-1^)	36.8	36.9	37.6
Vitamins (AIN-93M) (g.kg^-1^)	10.5	10.6	10.7
Cellulose (g.kg^-1^)	52.6	52.8	53.7
Choline (g.kg^-1^)	2.4	2.4	2.5

Total protein (% of energy)	20	20	20
Total carbohydrates (% of energy)	50	50	50
Total fat (% of energy)	30	30	30
Metabolizable energy (kcal.g^-1 ^of DM)	4.18	3.98	4.03

Indispensable AA (mg.g^-1 ^of protein)			
Valine	46	64	47
Tyrosine	35	46	31
Phenylalanine	48	46	46
Cysteine	15	9	22
Methionine	21	24	21
Lysine	60	76	63
Leucine	76	93	79
Isoleucine	45	58	38
Threonine	36	43	53
Tryptophan	13	13	14

Total	395	472	414

MPI: milk protein isolate; RPI: rapeseed protein isolate; OPI: other protein isolate.

All diets were moistened (powdered diet/water: 1:2) to prevent spillage. The food had the same consistency in all the groups. A feeding pattern designed to accustom rats to eating a standard meal in a short time, was applied: a first meal (6 g dry matter, representing approximately 33% of the daily intake) was given each day between 08:00 and 09:00 and then removed. Thereafter, the rats were allowed free access to food between 13:00 and 17:00. After the first two days of training, all animals ate all of the first meal within one hour. Both animals and their food consumption were weighed on four days each week.

### Experimental protocol

At the end of the 15-d dietary adaptation period, the rats were weighed and given the test meal (Figure 1). Within each of the four groups, half of the rats were used to measure fed-state tissue protein synthesis rates at 2 h after the meal and the other half were given a ^15^N-labeled MPI or RPI meal and killed 5 h after the meal in order to determine the postprandial fate of dietary N, as described below. In these latter groups, the body composition of the rats was also determined following a subcutaneous injection of deuterated water (60 mg.kg^-1 ^BW) 2 h prior to organ harvest.

The rats were anesthetized using an intraperitoneal injection of sodium pentobarbital (50 mg.kg^-1 ^BW, Sanofi Synthélabo Santé Animale, Libourne, France). After incision of the abdomen, heparinized blood was collected quickly from the vena cava and the animals were killed by rupture of the caudal vena cava and aorta. The blood samples were centrifuged for 15 min at 1500 g and 4°C, and the plasma was frozen at -20°C for subsequent analyses of plasma urea, proteins, AA and deuterated water.

Tissue protein synthesis rates were assessed using the flooding dose method as adapted by Mosoni *et al*. [13] Rats were injected subcutaneously with 300 μmol/100 g BW of a flooding dose of L-[1-^13^C]-valine (50 mol%, Cambridge Isotope Laboratories, Andover, MA, USA), 30 minutes prior to organ harvest. The intestinal mucosa, liver, kidneys and gastrocnemius muscle were promptly removed, rinsed, weighed, frozen in liquid N and kept at -20°C until analysis. A sample of hair-free skin was also collected and frozen at -20°C.

In order to measure the digestive and N metabolic fate of dietary N in tissues and urea, rats were anesthetized 5 h after ingesting the ^15^N-labeled meals. ^15^N-labeling of milk was performed as described previously [14]. Briefly, milk was collected from 3 lactating cows infused with ammonium sulfate (^15^NH_4_)SO_4 _(Eurisotop, St Aubin, France) in the rumen for 5-d. ^15^N-micellar casein (intact casein) was extracted by microfiltration and then purified through water diafiltration. All processes were carried out at the National Institute for Agricultural Research (UMR STLO, INRA Rennes, France). Meal enrichment was 0.5526 AT%. The urine was collected throughout the postprandial period, weighed and frozen at -20°C. The contents and walls of the different segments of the gastrointestinal tract were collected, weighed and frozen at -20°C, as were the liver, kidneys and gastrocnemius muscle. A sample of hair-free skin was also collected and frozen at -20°C.

### Analytical methods

Frozen tissues were pulverized in liquid N. Free and protein-bound AA were separated by precipitation with 5-sulfosalicylic acid (10%). After centrifugation (2500 g, 4°C, 20 min), the free AA fraction supernatant was extracted using cation exchange resin (AG 50W-X8 resin, H^+ ^form, 100-200 Mesh Hydrogen form, Bio-Rad, Hercules, CA), eluted with NH_4_OH 4 M and dried by evaporation. The pellet was rinsed and centrifuged three times with 1 mL 5-sulfosalicylic acid (10%), and then freeze-dried. Tissue proteins were hydrolyzed for 48 h at 110°C with HCl 6 N under N_2_, and the AA released were extracted using the same procedure as that described for the free pool.

To determine ^13^C-valine enrichment in free tissue AA pools, derivatization was ensured according to the silylation method: the AA extract (2 to 2.5 mg) was added with 80 μL acetonitrile (Acros Organics) and 80 μL N-tert-butyldimethylsilyl-N methyltrifluoroacetamide (Fluka), mixed and heated for 30 min at 110°C. The samples were kept in hermetic vials at -20°C until analysis. ^13^C-valine enrichments were determined using GC-MS (Hewlett-Packard 6890 N, Palo Alto, CA, USA) with electron impact ionization and selected ion monitoring of m/z 288 and 289 for the M+0 and M+1 ions, respectively. In tissue protein-derived AA, the same derivatization than free AA pools was used. ^13^C-valine enrichment was evaluated by GC-C-IRMS (HP5890/Isoprime, VG Instruments, Manchester, UK) using a 50 m apolar column (HP5MS, Hewlett-Packard). The ^13^C-enrichment of leucine was also determined and used as a proxy for the baseline tissue protein-bound ^13^C-valine enrichment of each individual animal [15]. Since it is not possible to assess the baseline enrichment in 13C-valine in the rats that receives the 13C-valine injection, the classical approach is to use for each rat an average value assessed from a separate subgroup of rats that have not been administered the injection. Alternatively, we chose to determine 13C-leucine enrichment in each rat that received the injection, as a proxy for its baseline 13C-valine enrichment. As compared to the classical method, the advantage is that the value is determined in each rat, but the drawback is to use leucine as proxy. A clear limitation is that we cannot ascertain that the present method does not convey an error that is higher than the classical method. However, the error, if any, is expected to be the same in all group and the conclusion of the present study would still remain valid.

The ^15^N-enrichment and N content in either the whole or in some N fractions of digesta, tissues, plasma and urine were measured by EA-IRMS after freeze-drying for digesta and tissues and N fractionation for plasma and urine. Before the freeze-drying process, tissues were incubated during 2 h at 80°C. In plasma, the urea, AA and protein fractions were separated. The protein fraction was extracted by addition of 50 μL 5-sulfosalicylic acid (100%) per mL of plasma. After centrifugation (2000 g, 20 min and 4°C), the protein pellet was freeze-dried. The supernatant was buffered at pH 7 and the urea fraction of the supernatant was extracted using cation exchange resin (AG 50W-X8 resin 100-200 Mesh, Na form, Bio-Rad, Hercules, CA) in the presence of urease (Sigma) for 2 h at 30°C. The resin containing the urea-derived ammonia from serum was washed with distilled water and stored at 4°C. Ammonia was eluted with KHSO_4 _(2.5 M) just before analysis. The remaining fraction of plasma AA was freeze-dried. In urine, the ammonia and urea fractions (after hydrolysis) were isolated on cation exchange resin (AG 50W-X8 resin 100-200 Mesh Na form, Bio-Rad, Hercules, CA). Before isotopic determination, the resins were eluted with KHSO_4 _(2.5 M). ^15^N-enrichment and N content were measured by isotopic ratio mass spectrometry (Optima, Fisons Instruments, Manchester, UK) coupled to an elemental analyzer (NA 1500 series 2, Fisons Instruments). Urea concentrations were determined using a commercial kit (Bio-Mérieux, Marcy l'Etoile, France).

### Calculations

Fractional synthesis rates (FSR, %/d) of tissue proteins were calculated as:

where E_bound val _and E_free val _are protein-bound and free ^13^C-valine enrichments in tissues (mol percent excess, MPE) and t the time (in h) elapsing between the ^13^C-valine injection and tissue collection. E_bound val _(MPE) was calculated as:

where E_protValine _and E_protLeucine _are protein valine and leucine enrichments, respectively, in MPE.

E_free val _(MPE) was calculated as:

where R_s _is the abundance of ion M+1 relative to ion M in the sample (after the administration of the tracer) and R_bk _(the "background" abundance), is the average abundance determined from a series of samples from separate control animals. Of note, because the flooding dose technique induces a dramatic increment in E_freeval_, one can rightfully neglect R_bk _as compared to R_s_.

Absolute synthesis rates (ASR, g.d^-1^) were calculated as:

where P is the tissue total protein content (in g).

Tissue percentage dry matter (%DM, g/100 g) was determined after freeze-drying of the tissue and served to calculate tissue hydration. %DM was calculated as:

where TM_dry _is tissue mass after freeze-drying (g) and TM is tissue mass (g).

The total N content (N_tot_, g) of tissues, gastrointestinal content, plasma and urine was determined as:

where TM refers to tissue mass (g) and %N to the percentage of N in dry tissue (g/100 g). The crude protein content was obtained by N × 6.25.

The incorporation of dietary N (N_exo _, % of ingested N) in tissues, gastrointestinal content, plasma and urine was expressed as a percentage of total N (N_tot_, mmolN):

where E refers to enrichment (Atom%) and N_ing _to ingested N (mmolN).

The real ileal digestibility (Dig, %) of protein vas determined as:

where ∑N_exo _is the amount of dietary N found in the intestinal, cecal and colon contents (mmolN) and N_ing-corr _is the amount of ingested N corrected for the amount still present in the stomach 5 h after the meal (4.8% on average).

Total body water (TBW, g) was determined as:

where D_2_O refers to the injected quantity of D_2_O (mg), E_D2O _the isotopic enrichment (Atom%) of injected deuterated water and E _basal _and E_plasma 2 h _the baseline and 2-h plasma water enrichments, respectively.

Fat free mass (FFM) was calculated as FFM = TBW × 0.736 [16], where 73.6% is the mean hydratation factor. Total muscle mass was not directly measured but was derived from the measured fat-free mass, considering that skeletal muscle mass represents 60% of fat-free mass [17].

The total deamination losses were calculated as:

where N_exo urine _refers to N incorporation in urine (mmolN), [plasma urea] the plasma urea concentration and E enrichment (Atom%).

The contribution (%) of dietary N to postprandial tissue protein synthesis was calculated for each individual by dividing the amount of N of dietary origin present in each tissue 5 h after the meal by the total amount of protein N synthesized during the same period. The latter was calculated using the amount of protein as measured in each individual and the average FSR value of the corresponding diet group, as assessed in the separate experiment:

### Statistics

Data are expressed as means ± standard deviation (SD). The effects of the protein source (PS: RPI or MPI), adaptation (A: acute or chronic) and their interactions (PS*A) were analyzed using 2-way ANOVA (SAS 9.1, SAS Institute, Cary, USA). Post-hoc Tukey tests for multiple comparisons were performed to enable pairwise comparisons. Differences were considered significant at *P *< 0.05.

## Results

During this study, we compared the postprandial protein metabolism profiles after the ingestion of RPI or MPI in rats. To test the effect prior adaptation to the protein source on the response, different groups of animals either received the protein sources for the first time (acute conditions) or after a 15-d adaptation period (chronic conditions).

### Influence of protein source during the adaptation period on the dietary intake, body weight and tissue composition of the rats

There were no overall significant differences between groups regarding their initial and final weights after 15 days of dietary adaptation (ANOVA, *P *= 0.07), although OPI rats displayed a significantly, slightly lower body weight gain than RPI rats (Table 2). Energy and protein intake did not differ between the groups. The food efficiency ratio (weight gain/energy ingested) of OPI rats was 13% lower than that of RPI rats. However, the sizes of the main body compartments (total water, fat-free mass, fat mass) were not influenced by the diet. By contrast, some tissue masses and protein contents differed slightly between groups: the stomach mass was lower in RPI rats when compared to the two other groups (-11%) and the kidney mass was higher in OPI rats than in MPI rats (+8%; Table 2). These variations in tissue mass were not accompanied by changes in protein content. Only the protein content of skeletal muscle (gastrocnemius) was higher in MPI and RPI rats than in OPI rats and protein content of cecum was higher in MPI rats than in OPI and RPI rats.

**Table 2:** Effect of adaptation diet (15 days) on growth, dietary consumption, body and tissue composition in rats (n = 48)

	OPI		MPI		RPI		
	
	Mean	SD	Mean	SD	Mean	SD	Stat effect*^a^*
**Weight**							
Initial weight (g)	210	9	210	6	209	8	NS
Final weight (g)	282	14	290	14	292	9	P = 0.07
Weight gain (g.d^-1^)	4.8^a^	0.7	5.3^ab^	0.7	5.5^b^	0.5	P < 0.02

**Consumption**							
Food intake (kcal.d^-1^)	74.5	5.3	74.2	4.2	74.8	6.0	NS
Protein intake (g.d^-1^)	3.7	0.3	3.7	0.2	3.7	0.3	NS
Food efficiency (g.kcal^-1 ^×100)	6.8^a^	0.9	7.5^ab^	1.0	7.9^b^	0.9	P < 0.005

**Body composition^*b*^**							
Total body water (g)	177	11	188	10	181	11	NS
Fat free mass (g)	243	15	257	13	248	15	NS
Fat mass (g)	42	19	29	8	39	22	NS
Fat mass (%)	14.5	6.2	10.2	2.8	13.6	7.5	NS

**Tissue mass (g/100 g BW)**							
Stomach	0.51^a^	0.03	0.52^ab^	0.05	0.46^b^	0.03	P < 0.0005
Intestinal mucosa	2.69	0.28	2.68	0.31	2.65	0.23	NS
Cecum	0.24	0.04	0.28	0.04	0.23	0.05	P = 0.08
Colon	0.36	0.05	0.35	0.04	0.35	0.04	NS
Liver	3.36	0.24	3.31	0.20	3.31	0.19	NS
Kidney	0.71^a^	0.04	0.66^b^	0.03	0.68^ab^	0.06	P < 0.05
Gastrocnemius muscle	0.183	0.008	0.180	0.008	0.179	0.008	NS
Total skeletal muscle *^c^*	51.6	1.9	52.4	3.1	50.3	1.9	P = 0.08

**Tissue protein content (mg)**							
Stomach	234	22	239	42	214	41	NS
Intestinal mucosa	867	92	841	100	812	98	NS
Kidney	366	32	360	35	357	37	NS
Liver	1957	163	2026	168	1996	156	NS
Colon	107	32	100	36	103	38	NS
Cecum	76^a^	14	95^b^	14	67^a^	8	P < 0.01
Gastrocnemius muscle	112^a^	8	120^b^	11	120^b^	8	P < 0.01
Skin	12841	1134	13455	1448	13644	1432	NS

NS: not significant, BW: body weight. *^a ^*One way ANOVA; *^b ^*Determined by deuterated water method; ^*c *^Estimated from fat-free mass (see Materials and methods). Means in a row with different letters are significantly different (P < 0.05, Tukey post-hoc test).

### Influence of protein source on dietary N digestibility and metabolic losses

Five hours after the meal, dietary N contents in the lumen of the stomach, cecum and colon did not differ significantly between groups (Table 3). More dietary N was found in the small intestinal lumen in RPI than in MPI rats, whatever the adaptation (*P*<0.001). Prior adaptation to the protein sources diminished the amount of dietary N recovered from the proximal colon. The true ileal digestibility of MPI and RPI were ~95% in all groups (NS), whether the animals were adapted to receiving the respective proteins or not. Five hours after the meal, a significantly (*P*<0.05) higher proportion of plasma AA of dietary origin was found in MPI rats than in RPI rats (ratios of 0.18 ± 0.04 and 0.17 ± 0.06 for MPI under acute and chronic conditions, respectively and 0.13 ± 0.02 and 0.14 ± 0.01 for RPI under acute and chronic conditions, respectively). Losses (digestive, deamination) of dietary N were not influenced by either the protein source or by adaptation (Table 3).

**Table 3:** Recovery in the lumen of the gastro-intestinal compartments, digestibility and losses of dietary nitrogen in rats 5 h after the meal

	Acute				Chronic				
	
Dietary nitrogen	MPI		RPI		MPI		RPI		
**(% of ingested) in:**	**Mean**	**SD**	**Mean**	**SD**	**Mean**	**SD**	**Mean**	**SD**	**Stat effect *^a^***

Stomach	5.23	4.59	3.02	2.14	5.28	5.48	5.32	4.83	NS
Intestine	1.62^ab^	0.51	2.18^a^	0.23	1.32^b^	0.13	2.32^a^	0.75	PS: P < 0.001
Cecum	2.08	0.47	1.61	0.50	2.20	1.11	1.92	0.38	NS
Proximal colon	0.67	0.49	0.56	0.31	0.21	0.16	0.40	0.18	A: P < 0.05
Distal colon	0.15	0.20	0.41	0.46	0.28	0.52	0.21	0.24	NS
Digestives losses ^*b*^	2.9	0.7	2.6	0.6	2.7	1.2	2.5	0.5	NS
Ileal digestibility *^c^*	95.2	1.0	95.1	0.5	95.8	1.1	94.9	1.1	NS
Deamination ^*d*^	11.4	1.7	11.7	3.2	13.2	2.9	14.1	5.0	NS
Total losses	14.4	1.9	14.2	3.3	15.9	2.7	16.6	5.3	NS

NS: not significant, PS: protein source, A: adaptation. ^*a *^Two way ANOVA; *^b ^*Recovery in cecum and colon content at 5 h (% of ingested N); ^*c *^Real ileal digestibility (see Material and methods); *^d ^*Recovery in body and urinary urea. Means within a row with different letters are significantly different (P < 0.05, Tukey post-hoc test)

### Postprandial metabolic retention of dietary N in tissues and body N pools

**Figure 2:**
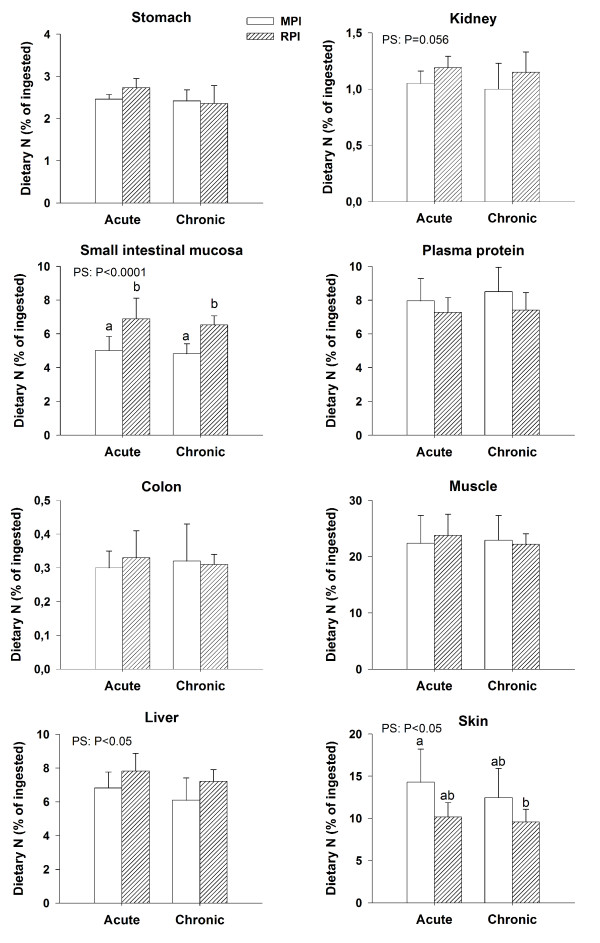
**Dietary nitrogen (N) incorporation into tissues of rats 5 h after the meal**. The meal was based on milk protein isolate (MPI) or rapeseed protein isolate (RPI) ingested for the first time (acute) or after prior 15d-adaptation to this protein source (chronic). Results are expressed as means ± SD, each bar represents 6 rats. Statistical effects are reported from an ANOVA with protein source (PS) and adaptation (A) and their interaction PS*A as factors. Bars with different letters are significantly different (*P*<0.05, Tukey post-hoc test).

Dietary N incorporation into tissues was differently influenced by the protein source (MPI or RPI) as a function of tissues, whereas adaptation (acute or chronic) had no effect (Figure 2). The ingestion of RPI, as compared to MPI, elicited a higher incorporation of dietary N in the small intestinal mucosa (+36% for pooled acute and chronic values, *P*<0.0001) and liver (+16%, *P*<0.05), with a trend towards a higher incorporation in kidneys (+14%, *P *= 0.056). MPI ingestion was associated with a higher incorporation of dietary N into skin (+36%, *P*<0.05) as compared to RPI. The pattern of dietary N distribution in body N pools at the end of the postprandial period is shown in Table 4. When averaged among the groups, the regional accretion of dietary N was 25% and 35% of the ingested dose in visceral and peripheral organs, respectively. The incorporation of dietary N in all gastrointestinal tissues was significantly enhanced in RPI-fed rats when compared to MPI-fed rats (+24%, *P*<0.0001). The incorporation of dietary N in visceral organs was also greater (+9%, *P*<0.05) in RPI than in MPI rats. The recovery of dietary N in peripheral organs and in all body N pools did not differ between groups. Prior adaptation to the protein source had no effect on any tissue N retention parameters.

**Table 4:** Summary of the postprandial incorporation of dietary nitrogen into tissue in rats 5 h after the meal

	Acute				Chronic				
		
Dietary nitrogen	MPI		RPI		MPI		RPI		
		
(% of ingested) in:	Mean	SD	Mean	SD	Mean	SD	Mean	SD	Stat effect *^a^*
GI tract ^*b*^	7.9^a^	0.9	10.1^b^	1.2	7.8^a^	0.5	9.4^b^	0.9	PS: P < 0.0001

Hepatic pools ^*c*^	14.8	1.8	15.1	1.8	14.8	2.3	14.6	1.4	NS

Visceral organs ^*d*^	23.8	2.5	26.5	2.8	23.6	2.5	25.2	1.6	PS: P < 0.05

Peripheral pools ^*e*^	36.7	7.5	34.0	3.6	35.4	7.4	31.8	2.8	NS

Total incorporation	60.4	7.8	60.4	6.2	59.0	9.5	56.9	4.1	NS

NS: not significant, PS: protein source, A: adaptation. *^a ^*Two way ANOVA; *^b ^*GI: gastrointestinal, Incorporation in stomach, small intestine, cecum and colon; *^c ^*Incorporation in liver and plasma protein; *^d ^*Incorporation in hepatic pools, GI tract and kidneys; *^e ^*Incorporation in muscle and skin. Means in a row with different letters are significantly different (P < 0.05, Tukey post-hoc test).

### Postprandial tissue protein synthesis rates and the contribution of dietary N to postprandial protein deposition

The FSR and ASR of the major tissues involved in protein metabolism were not significantly influenced by the protein source or by prior adaptation (Table 5). Total protein synthesis, and visceral and peripheral organ ASR values were not significantly affected by the protein source or adaptation.

**Table 5:** Fractional synthesis rate (FSR) and absolute synthesis rate (ASR) of tissue proteins in rats after ingestion of the meal

	Acute				Chronic				
		
	MPI		RPI		MPI		RPI		Stat
		
	Mean	SD	Mean	SD	Mean	SD	Mean	SD	effect*^a^*
**Intestinal mucosa**									
FSR (%/d)	313	107	335	106	433	214	352	85	NS
ASR (gP/d)	1.36	0.56	1.45	0.55	1.80	1.03	1.46	0.29	NS

**Liver**									
FSR (%/d)	109	10	100	13	96	11	108	13	NS
ASR (gP/d)	2.06	0.26	1.97	0.37	1.87	0.29	2.22	0.36	NS

**Kidney**									
FSR (%/d)	81	12	78	19	93	36	81	22	NS
ASR (gP/d)	0.30	0.03	0.27	0.05	0.34	0.15	0.28	0.09	NS

**Total muscle**									
FSR (%/d)	9.7	2.1	9.5	2.7	11.3	4.0	10.9	2.1	NS
ASR (gP/d)	2.70	0.62	2.72	1.04	3.43	1.22	3.33	0.69	NS

**Skin**									
FSR (%/d)	17.0	5.3	21.3	4.0	24.4	9.6	16.8	6.5	NS
ASR (gP/d)	2.18	0.63	3.04	1.10	2.84	1.14	2.40	1.07	NS

Visceral organs*^b ^*(gP/d)	3.4	0.6	3.3	0.8	3.7	1.2	3.5	0.5	NS
Peripheral tissue*^c ^*(gP/d)	5.2	1.0	6.0	1.5	6.6	1.2	6.0	1.3	NS
Total protein synthesis (gP/d)	8.6	1.4	9.1	1.8	10.1	2.2	9.5	1.4	NS

NS: not significant. ^*a *^Two way ANOVA; *^b ^*Amount of intestinal mucosa, liver and kidney ASR; *^c ^*Amount of muscle and skin ASR.

**Figure 3:**
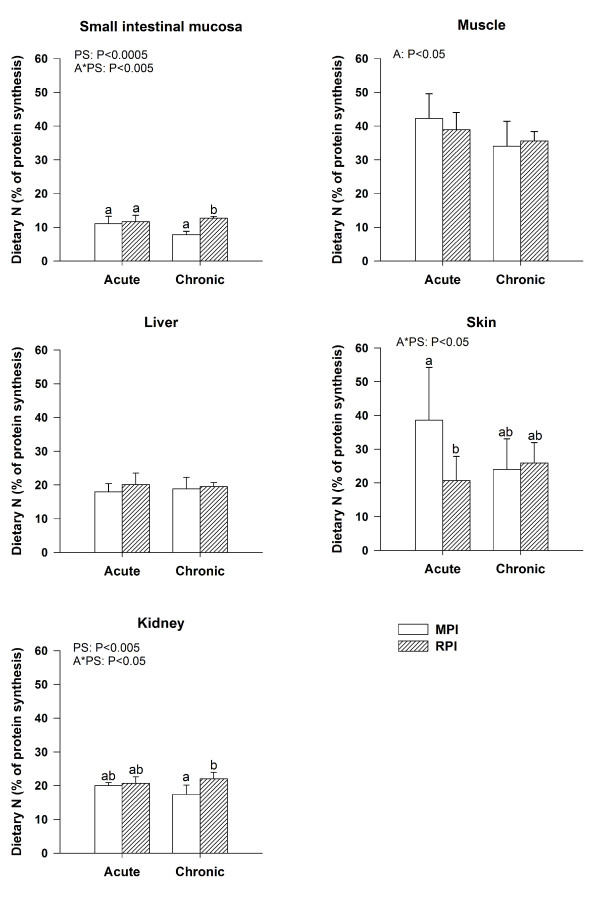
**Dietary nitrogen (N) contribution to the postprandial tissue protein gain in rats 5 h after the meal**. The meal was based on milk protein isolate (MPI) or rapeseed protein isolate (RPI) given for the first time (acute) or after prior 15d-adaptation to this protein source (chronic). Results are expressed as means ± SD, each bar represents 6 rats. Statistical effects are reported from an ANOVA with protein source (PS) and adaptation (A), and their interaction PS*A as factors. Bars with different letters are significantly different (*P*<0.05, Tukey post-hoc test).

Using the data obtained on postprandial N retention and FSR in tissues, we calculated the contribution of dietary N to the protein synthesized in each tissue during the postprandial period. The contribution of dietary N to postprandial protein deposition differed markedly between tissues (Figure 3). In the intestinal mucosa and kidneys, 63% and 27% (respectively) more dietary N was used for protein synthesis in RPI rats than in MPI rats under chronic conditions (P < 0.005). By contrast, the contribution of dietary N to skin postprandial N gain was significantly smaller in RPI rats than in MPI rats under acute, but not chronic, conditions. The contribution of dietary N to postprandial protein deposition in muscle was significantly higher under acute than under chronic conditions, whatever the protein source.

## Discussion

The present study revealed that despite very similar overall indices of postprandial dietary N digestion and retention *in vivo *in rats, the ingestion of rapeseed and milk protein isolates led to marked regional differences in dietary N utilization: RPI ingestion resulted in a greater retention of N in visceral organs whereas MPI enhanced that in the skin. By contrast, the FSR values of corresponding tissues were not influenced by the protein source. Finally, most of the differences arising between RPI and MPI postprandial metabolism were observed following the first ingestion of each protein source and persisted after adaptation.

Both RPI and MPI were characterized by a high true protein digestibility of 95% in rats. The value obtained during the present study is consistent with a published estimate of 95% for rapeseed protein digestibility in rats, within the same range as soy protein and slightly lower than casein [18]. We did not observe in this species the low true protein digestibility of RPI that we [4] and others [8,19,20] had previously evidenced in humans or other monogastric species. Rats may benefit from more efficient enzymatic equipment to digest hydrolysis-resistant rapeseed proteins than humans or pigs, and as such, are probably not a good model to study dietary protein digestibility in humans, unlike the pig [9,21], at least for dietary proteins with relatively slight differences in digestibility. In addition, RPI also did not differ from MPI in terms of postprandial metabolic losses of dietary N resulting from the deamination of dietary AA and excretion in urine. This indicates that RPI is a vegetable protein with a high biological value, which is consistent with other reports in humans and pigs [4,8]. Furthermore, we observed a good alignment of postprandial deamination values in rats (10-12% of ingested N/5 h) and in humans (12% of ingested N/8 h) [4]. Finally, our results indicated that the digestibility and postprandial retention of dietary N did not differ in rats between RPI and MPI, a result that is in line with the similar growth rate and final body composition observed in animals fed with either protein, and with previous reports showing that rapeseed protein was the vegetable protein with the higher nutritional value for rats [22].

Interestingly, when examining the partitioning of postprandial dietary N retention between tissues, important differences were observed between RPI and MPI-fed rats. When compared with the ingestion of MPI, that of RPI was associated with greater retention in visceral organs (small intestinal mucosa, liver and kidneys), at the expense of its retention in skin. These results confirm those obtained by analysis using a previously developed compartmental model [11] of the data collected in humans after the ingestion of a bolus meal containing ^15^N-labeled rapeseed proteins, where we found splanchnic and peripheral accretions of dietary N that reached 57% and 12% of the dose ingested, *vs*. 41% and 20% for milk proteins (Fouillet *et al*. unpublished results). In peripheral tissues, it was interesting to observe the much higher sensitivity of skin to dietary protein source when compared to muscle, as previously seen during other manipulations of dietary protein intake [15,23]. Moreover, in order to test whether the differential effect of RPI and MPI on the regional retention of dietary N during the postprandial phase could result from diet-induced modulations of protein turnover, this study also examined the postprandial rates of tissue protein synthesis. These were strikingly similar between diets, suggesting that the modulation of postprandial dietary N gain by the protein source did not result from a differential effect on protein synthesis. Therefore, the most likely hypothesis is that the pattern of postprandial dietary N gain mainly results from regional changes in protein degradation or a differential utilization between RPI and MPI of the dietary *vs*. endogenous AA for protein synthesis in specific tissues. Similarly, in humans, although milk and soy protein differently affect the regional partitioning of dietary N postprandial accretion [12] and differently promote lean tissue mass in exercising subjects [24], their differential effects on muscle protein synthesis were not directly evidenced [25]. By contrast, it has been reported that a diet rich in vegetable protein results in a weaker inhibition of postprandial protein degradation in humans, when compared to a diet rich in animal proteins [26]. A specific enhancing effect of lentils or beans protein on small and large intestine masses, protein content and fed state protein synthesis rates has been reported in the literature [27,28]. In humans, a reduction in albumin synthesis and plasma albumin levels occurs when vegetable protein consumption increases [29]. Lower muscle mass and protein synthesis rates have also been demonstrated with legume-based diets (lentils, beans or peas) versus casein in animals [28,30-32]. The reasons for these tissue effects are mostly unclear, in particular because the effect of dietary plant protein may be confounded in part by the potential digestive or metabolic effects of a series of associated factors (e.g. antinutritional factors, starch, indigestible polysaccharides). Although we also observed a higher anabolic utilization of RPI in the gastro-intestinal tract when compared to MPI, it is difficult to generalize such an effect to all vegetable proteins. Our results warrant further studies to determine the effects of vegetable protein sources on postprandial protein degradation, particularly at the tissue level.

The mechanisms responsible for the differences in the metabolic effects of RPI and MPI remain unclear. The kinetics of intestinal delivery are important modulators of postprandial protein metabolism [33,34] and directly affect the tissue distribution of dietary N [11,35]. We observed a lower incorporation of dietary N in plasma AA 5 h after the ingestion of RPI when compared to MPI, although in humans the kinetics of dietary N appearance in plasma AA have been found to be comparable for milk and rapeseed proteins [4,34]. Although the pattern of dietary N recovery in the lumen of different gastro-intestinal tract segments suggested similar digestive kinetics for the protein sources, there may have been some differences between MPI and RPI-fed rats regarding dietary AA intestinal delivery. Hormonal changes induced by a bean-based diet *vs*. milk protein-based diet have been related to changes in muscle protein turnover, probably resulting from other ingredients present in the bean diet, such as starch [31]. Hormonal factors were unlikely to account for the metabolic differences that we observed between RPI and MPI-fed rats because we used protein isolates with a high protein content (>80%) and carefully equilibrated all the other ingredients in the diets. Most probably, the differences between RPI and MPI arose from their different AA patterns. The promoting effect of RPI on dietary N accretion in the small intestinal mucosa might be related to its ~25% higher content in threonine, an indispensable AA that is utilized largely by this tissue for the production of mucins [36]. It has been reported that circulating threonine levels in rats are considerably higher after the ingestion of a high-fat meal containing RPI than of the same meal containing MPI [5]. Another important difference between RPI and MPI is their content in branched-chain AA (BCAA), which are 25% less abundant in RPI. It is well known that BCAA could modulate both synthesis and degradation [37]. However, although this hypothesis could explain the differential impact of RPI and MPI on skin protein metabolism, it does not explain the lack of difference on muscle or other tissues. Finally, and most importantly, the higher sulfur AA intake in RPI-fed rats may have played a role in the metabolic differences observed during our study. Plasma cysteine and methionine levels displayed sharp postprandial increases in RPI-fed rats when compared to MPI [5], suggesting a much higher intestinal delivery of sulfur AA after RPI, consistent with the high content in dietary protein. As methionine and cysteine are extensively used by the gut as precursors for the synthesis of protein or other important molecules [38,39], the high level of sulfur AA in RPI could explain the higher retention of dietary N in this tissue.

Of note, we found that the effects of the dietary protein source was almost irrespective of whether the rats received the protein for the first time or had received it in its diet for two weeks. The only differences were a short delay in dietary N recovery from the colon of rats chronically adapted to RPI or MPI, and some limited, fragmented effects on the contribution of dietary N to postprandial tissue protein accretion. These effects are likely due to the less optimum AA composition of the OPI diet that may marginally have affected the growth rate, induced a significantly higher kidney mass and decreased muscle protein content, when compared to MPI-fed rats. The similarities of digestive kinetics, regional tissue distribution and dietary N losses under acute or chronic conditions of consumption of the two protein sources suggest that these parameters initially responded to the immediate effect of dietary protein after ingestion and were not driven by any phenomenon related to metabolic regulation, which would occur after a chronic consumption of these protein sources. This finding lends further credence to the findings of studies on the postprandial metabolism of meal protein under acute condition [4,40-42]. Although obtained in two series of experiments, we could combine data on dietary N distribution and regional protein synthesis rates to estimate the contribution of dietary N to the amount of protein deposited in each tissue, which, to our knowledge, constitutes a novel finding. These contributions reached 11% for the small intestinal mucosa, 19% for the liver, 20% for the kidneys, 27% for the skin and 38% for the skeletal muscle. This result might be considered as counter-intuitive, inasmuch as the contribution of dietary N was thus inversely related to the turnover of these tissues, and also to their "distance" from the site of dietary N absorption. This also means that the tissues the most strongly impacted by the dietary N supply were the muscle and skin. We believe that this original finding constitutes an interesting contribution to general knowledge of postprandial protein metabolism. It should however be noted that there were some differences between the protein diets: RPI-derived N contributed more to protein deposition in the small intestine and kidneys (after adaptation), while under acute conditions, rats receiving MPI displayed twice as much incorporation of dietary N in skin protein as RPI-fed rats.

## Conclusions

We have shown that protein sources of good nutritional value, and which enable similar growth and tissue composition, exert a markedly different impact on regional tissue protein metabolism during the postprandial period. We strongly suspect that the indispensable AA pattern of the protein was responsible for these differences, thus emphasizing the specificity of RPI as a protein source that can induce particular metabolic effects at the tissue level in healthy individuals. The mechanisms and functional consequences of these effects now warrant further study.

## Abbreviations used

RPI: rapeseed protein isolate; MPI: milk protein isolate; FSR: Fractional synthesis rates; N: Nitrogen; OPI: other protein isolate; AA: amino acid; BW: body weight; GC-MS: gas chromatography-mass spectrometer; GCC-IRMS: gas chromatography combustion-isotopic ratio mass spectrometer; EA-IRMS: elemental analyzer-isotopic ratio mass spectrometer; ASR: absolute synthesis rate; DM: dry matter; TM: tissue mass; E: enrichment; TBW: total body water; SD: standard deviation; PS: protein source; A: adaptation.

## Competing interests

The authors declare that they have no competing interests.

## Authors' contributions

The authors' responsibilities were as follows: BC. performed the experiments and analysis and participated to data interpretation and manuscript writing. FH. and MF. contributed to study design, data interpretation and manuscript writing. BF. and TD. contributed to the study design and manuscript writing. BC. designed the study, participated in experimental work, data interpretation and manuscript writing. All authors read and approved the final manuscript.

## Aknowledgements

This work was supported by funds from the National Institute for Agricultural Research (INRA) and by grants from the Technical Center for Oilseed Crops (CETIOM: Centre Technique Interprofessionnel des Oléagineux Métropolitains, Pessac, France) and from the National Agency for Oilseeds Development (ONIDOL: Organisation Nationale Interprofessionnelle des Graines et Fruits Oléagineux, France). We wish to thank Evelyne Fénart (ONIDOL) and Jacques Evrard (CETIOM) for their support on this project. There was no conflict of interest.
